# Polymorphism of floral type gene *Cly1* and its association with thermal stress in barley

**DOI:** 10.1371/journal.pone.0193390

**Published:** 2018-03-01

**Authors:** Meilin Zou, Gaofeng Zhou, Tefera Tolera Angessa, Xiao-Qi Zhang, Chengdao Li

**Affiliations:** 1 Western Barley Genetics Alliance, Murdoch University, Murdoch, Australia; 2 Western Australian State Agricultural Biotechnology Centre, Murdoch University, Murdoch, Australia; University of Tasmania, AUSTRALIA

## Abstract

Cleistogamy refers to a type of sexual breeding system with closed flowers. Cleistogamous flowers shed their pollen before flower opening, which leads to autogamy. Two SNPs in the open reading frame region of the *Cly1* gene are associated with floral type. In the present study, we investigated the floral type of 436 barley accessions. Molecular markers were developed to genotype these barley accessions based on the two SNPs in the *Cly1* gene region. The molecular markers explained floral type in 90% of the accessions. The *Cly1* gene was sequenced in accessions with inconsistent genotype and phenotype. Thirteen SNPs were detected with ten new SNPs in the gene region. We further investigated whether floral type was associated with temperature stress tolerance in four field trials. One site experienced frost stress with a minimum temperature of -3.4°C during flowering. Grain fertility rates as low as 85% were observed at this site but ranged from 92–96% at the other three sites. The relationship between grain fertility rate and floral type under temperature stress was inconclusive. Some lines with higher grain fertility rates were identified under frost stress, and would be useful for frost stress studies in barley.

## Introduction

Temperature stress is a significant obstacle to barley production. Temperature critically defines crop plant flowering time and development [1]. There is an apparent negative correlation between crop yield and elevated temperature, especially in barley, maize, and wheat [2]. Higher temperatures, also called heat stress, significantly affect floret growth rate during anthesis and heading [3]. Heat stress can reduce grain filling percentage and thousand-grain weight. The swelling of pollen grains can be severely inhibited by high temperature at flowering [4]. Frost stress, caused by very low temperatures, is also an obstacle to crop production. Losses in barley due to frost damage during flowering in Western Australia were estimated at $140 million in 2016 (http://www.giwa.org.au). Recently, a study found that cleistogamy reduced the effect of heat stress during flowering in rice [5].

Cleistogamy is defined as a type of sexual reproduction system where pollination and fertilization processes take place in an unopened flower [6]. In contrast, chasmogamy is where these processes take place in opened flowers [6]. Both cleistogamy and chasmogamy have been used to describe plant species in taxonomy. Cleistogamy is an adaptive mechanism for maintaining reproduction in unfavorable conditions, such as airflow and sunlight [7,8]. Cleistogamous plants are relatively widespread in 693 angiosperm species, across 228 genera and 50 families [9]. It predominantly appears in invasive species and annual plants [10]. Cleistogamy protects plants from a series of fungal infections [10] and decreases pollen-mediated gene exchange that benefits genetic uniformity [10,11,12,13,14,15,16]. Chasmogamous plants exhibit self-pollination or cross-pollination, resulting in more variance in the progeny.

Although the concept of cleistogamous flowers was first put forward by Kuhn in 1867 [9], the genetic control and molecular mechanisms of cleistogamy remained unknown for some time. There have been recent reports of breakthroughs in the occurrence and regulatory mechanisms of cleistogamy in plants [4,5,17,18,19,20,21].

Barley (*Hordeum vulgare* L.) exhibits both cleistogamous and chasmogamous flowers [10,18]. In cleistogamous barley, the closed palea and lemma are tightly associated with lodicule atrophy [10,18]. Morphologically, cleistogamous flowers have smaller lodicules than chasmogamous flowers [18]. A segregation analysis indicated that lodicule size co-segregates with flowering type [18]. Cleistogamous and chasmogamous barley types had significant differences in lodicule size at the early/white anther stage [10], possibly due to very active cell division in the chasmogamous type. At the later/green anther stage, the chasmogamous type had lodicules about twice the size of the cleistogamous type [10].

Genetically, cleistogamy is controlled by one or two dominant or recessive genes in important crops, such as sorghum, rice, wheat, soybean and barley [16,18,22,23,24,25]. In barley, a dominant gene *Cly2* affects a recessive gene *cly1* that governs cleistogamy [10,16,18,21,26].

One study used two F2 barley populations, derived from Azumanmugi × Kanto Nakate Gold and OUH602 × KNG, to fine-map the cleistogamy gene*cly1*to a 0.66 cM region on chromosome 2H [10]. Comparing the relevantsynety sequence in rice revealed a gene encoding an AP2 protein. This protein contains 487 residue polypeptides with two AP2 domains [10]. The second AP2 domain is the miR172 target region, with a single nucleotide difference between the two cultivars AZ and KNG [10]. By sequencing this gene in 274 barley lines, two SNPs in the target region were associated with cleistogamy [10].

In the present study, we developed molecular markers for the *Cly1*gene, genotyped 436 barley accessions, and identified the haplotypes of the *Cly1* gene based on sequencing results. The relationship between floral type and thermal stress is also discussed.

## Materials and methods

### Materials

This study evaluated 436 accessions including Australian barley varieties, advanced breeding lines, andgenotypes from Africa, Asia, Europe, North America, South America, and the International Centre for Agricultural Research in the Dry Areas (ICARDA). In 2016, the barley accessions were grown in four experimental fields (Geraldton, Esperance, Merredin, and Katanning) across Western Australia (S1 Fig). Geraldton is in the north of Perth with lower rainfall while Esperance is in the south southeast of Perth with high rainfall. The other two trials are located between Geraldton and Esperance with medium rainfall and extreme temperatures. The field trial was planted in a randomized complete block design with 1 × 3 m^2^ plots. Control varieties were used for spatial adjustment of the experimental data based on the Best Linear Unbiased Prediction (BLUP) [27].

### Development of molecular markers

Allele-specific PCR was used to detect single nucleotide polymorphisms (SNP) variants. The principle is to identify mismatches within the 3’ end of the allele-specific primers [28,29,30,31,32,33,34]. According to Nair et al. [10], two SNPs (SNP11 and SNP12 in this study) in the *Cly1* gene region (GCAGC[A/G]TCATC[A/C]) were associated with cleistogamy. Two sets of primers were developed for each SNP, and the results of the two sets were complementary. The primers used in the present study are listed in Table 1 and S2 Fig.

**Table 1:** Primers used in this study.

Name	Direction	Sequence	
U1488	Forward	AGAGAGGCCGATAGGGGTGGA	Amplify *Cly1* gene
U500	Forward	CGTTCCCGCAGCTCGCAGTAC	Amplify *Cly1* gene
U56774	Forward	CAGGGATTGCGGCAAGCAGGT	Amplify *Cly1* gene
L841	Reverse	ACCTGCTTGCCGCAATCCCTG	Amplify *Cly1* gene
L691	Reverse	CATGTGGGGGGCTTGCAGTTG	Amplify *Cly1* gene
SNP11Fa	Forward	CCGCAGCAGCAGCAGCATCG	Specific primer for one SNP with SNP11R
SNP11Fb	Forward	CCGCAGCAGCAGCAGCATCA	Specific primer for one SNP with SNP11R
SNP11R	Reverse	GCTGGTAATGGCTGTGGGACG	Reverse primer for one SNP
SNP12F	Forward	ACCAGCAGCAGCAACAGAGGC	Forward primer for another SNP
SNP12Ra	Reverse	TGGCGATGTAGGGTGGGAATAGG	Specific primer for another SNP with SNP12F
SNP12Rb	Reverse	TGGCGATGTAGGGTGGGAATAGT	Specific primer for another SNP with SNP12F

### PCR and sequencing

PCR amplification was carried out in a total volume of 10 μl that contained: 1 μl Bioline 10× reaction buffer, 0.3 μl 50 mM MgCl_2_, 0.2 μl 10 mM dNTP, 0.2 μl 10 μM primers, 1 unit Taq polymerase (BIOTAQ DNA polymerase), and 1 μl 100 ng/μl DNA template. The PCR program was 95°C for 4 min, followed by 34 cycles of 94°C for 30 s, 56°C for 30 s, and 72°C for 30 s, then 72°C for 5 min and kept at 14°C. PCR products were run on 2% electrophoresis agarose gel. For sequencing, PCR products were excised from agarose gel. The excised gel was placed into a 200 μl filter tip and transferred to a 1.5 ml microcentrifuge tube. The cusp part of the tips was cut to fit the collection tube. The tube was centrifuged for 30–60 s at 14,000 rpm. The pipette tip was then removed, and the PCR fragments in the tube were used for the sequencing reaction.

The sequencing reaction consisted of 1 μL Big Dye, 1.5 μL reaction buffer, 0.5 μl of 5 μM primer, and 2 μL purified PCR product DNA and made up to 10 μl with sterile distilled water. The sequencing reaction was processed under the following conditions: 95°C for 2 min, 25 cycles of 95°C for 10 s, 50°C for 10 s, and 60°C for 4 min, and then kept at 25°C.

The reaction product was transferred to separate 0.5 ml centrifuge tubes. A mixture of 3 μl of 3 M sodium acetate and 25 μl of 100% ethanol was added to each centrifuge tube for DNA precipitation. The sample was mixed and stood at room temperature for 15 min. The tubes were centrifuged at maximum speed (14,000 rpm) for 15 min. The supernatants were gently removed by pipette. The pellets of DNA at the bottom of each centrifuge tube were washed with 30 μL of 70% ethanol. Subsequently, the samples were centrifuged at maximum speed (14,000 rpm) for 5 min. Finally, the wash solution was removed, and the pellet dried at room temperature. The samples were ready for sequencing. The sequences for the barley cultivars, Morex, Bowman, and Barke were downloaded from http://webblast.ipk-gatersleben.de/barley_ibsc/. The software Geneious 6.0 was used to analyze the sequence.

### Phenotyping

Following the appearance of the first awn (Zadoks’ growth stage 49) in the earliest flowering genotypes, the trial was regularly visited and the flowering type data recorded.

The flowering type of an accession was recorded as chasmogamous (open flower type) if the stamen (anther and filament) of at least one plant in a plot extruded from its spike (S3 Fig). But if the stamen did not extrude from the spike, we investigated the gaps between leman and palea. If the gap was closed, the plants were recorded as cleistogamous, but if the gap was open, the plants were recorded as chasmogamous even though the stamen was still inside. Genotypes without chasmogamy at any site visit were recorded as cleistogamous flower type. At maturity, 50 heads were randomly harvested from each plot. Total grain numbers and sterile spikelets were counted.

### Weather data collection

The weather data, temperature, and relative humidity were recorded using Tinytag, Gemini Data Loggers (UK) Ltd (www.tinytag.info). Two Tinytags were installed at each trial site at two different heights: (1) 0.5 m to record within canopy weather data; (2) 1.5 m to measure aerial weather data. The Tinytags recorded the weather data at 15 min intervals throughout the season.

### Statistical analysis

Association analysis between floral type and grain fertility rate was conducted for each site. Since most of the barley accessions were chasmogamous, we randomly selected chasmogamous accessions with similar flowering times to the cleistogamous accessions. Correlation coefficients were calculated in Microsoft Excel. A*t*-test was performed to determine any significant differences (*p≤*0.05) in grain fertility between cleistogamous and chasmogamous barley.

## Results

### Phenotyping

The flowering types of the 436 barley accessions were surveyed in four field trials. Of these accessions, 372 were the chasmogamous type, and 64 the cleistogamous type. In other words, the closed flowering type accounted for only 14.7% of the barley accessions in this study.

### Genotyping

**Fig 1:**
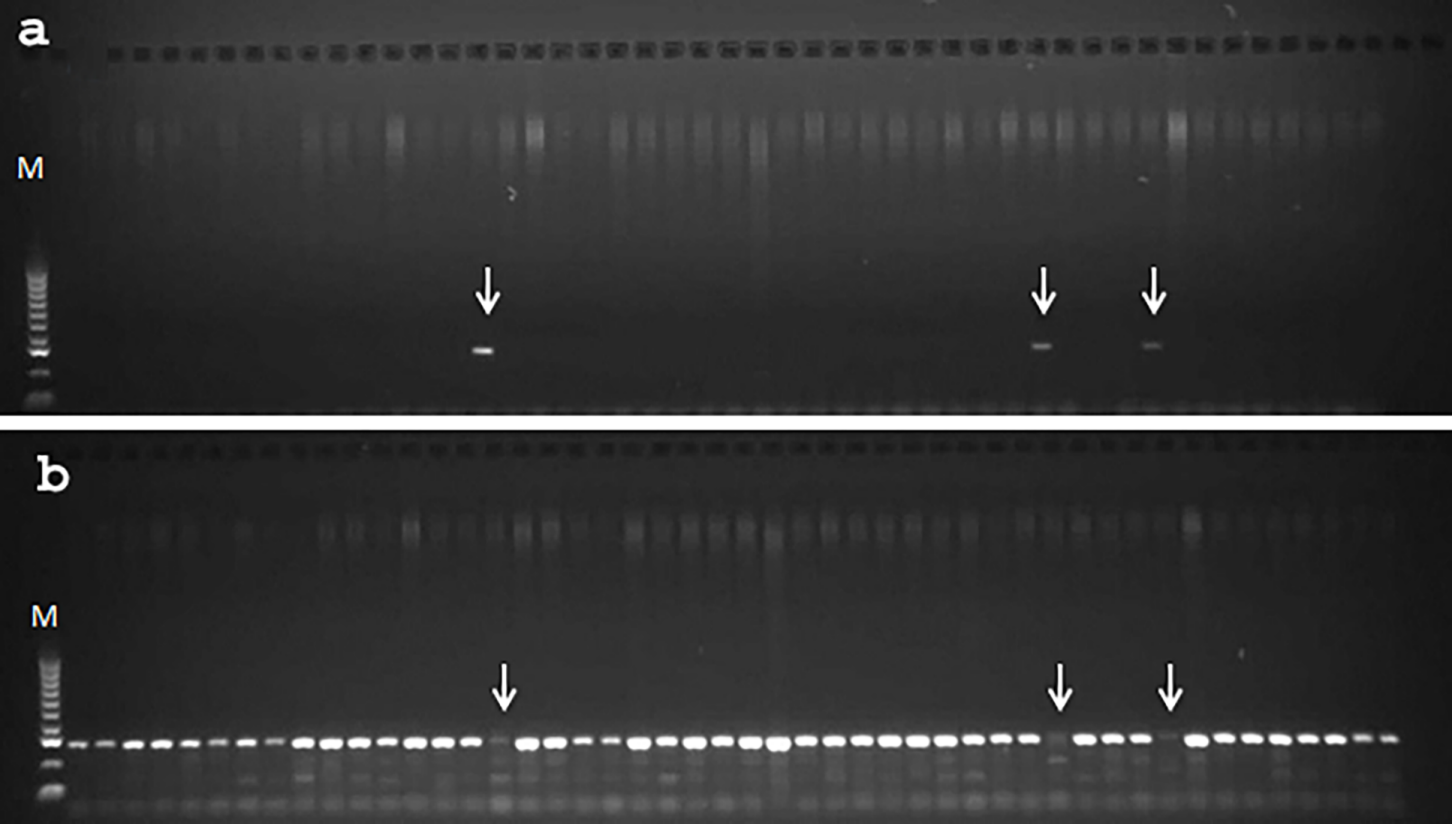
Genotyping floral type with molecular markers. (a) Using a molecular marker to amplify the cleistogamous type. (b) Using a molecular marker to amplify the chasmogamous type. Arrow indicates PCR products from cleistogamous types. M stands for 100 bp DNA ladder.

Allele-specific markers were developed from the two SNPs associated with flowering type in the *Cly1* gene region. Fig 1 is an example of how the markers worked. Fig 1A shows that three of 48 accessions were amplified with a marker that amplifies the cleistogamous type. A marker that amplifies the chasmogamous type was used to confirm this result (Fig 1B), such that the same three accessions were not amplified. We can conclude from Fig 1 that three accessions have a cleistogamous genotype and 45 have a chasmogamous genotype. The genotypes of the 436 barley accessions were characterized using this method, such that 29 accessions were cleistogamous and 407 were chasmogamous.

Association analysis of the genotype and phenotype of these accessions identified 391 samples with consistent genotype and phenotype for flowering type. Of these, 367 were the chasmogamous type and 24 the cleistogamous type. The new molecular markers explained the variation in floral type in 90% of the barley accessions. The remaining 45 lines had inconsistent genotype and phenotype and were divided into two groups. The first group contained five barley lines (Kinukei7, Langstrand0102, C2-05-101/263, Prosa, WVB 35) with cleistogamous genotype and chasmogamous phenotype. The second group contained 40 barley lines with chasmogamous genotype and cleistogamous phenotype (Table 2).

**Table 2:** The genotypes and phenotypes of 45 lines. The genotypes of SNP11 and SNP12 were expressed as A, G and C. CL stands for cleistogamous type, while N stands for chasmogamous type.

Name	SNP11	SNP12	Genotype	Phenotype
C2-05-101/263	G	A	CL	N
Kinukei 7	G	A	CL	N
Langstrand 0102	A	C	CL	N
Prosa	A	C	CL	N
WVB35	G	A	CL	N
04S213D-3-125	A	A	N	CL
Arapiles	A	A	N	CL
B511	A	A	N	CL
BEARPAW	A	A	N	CL
BM9647D-43	A	A	N	CL
Bmnl-28	A	A	N	CL
BOLRON	A	A	N	CL
Buloke	A	A	N	CL
C2-05-101/437	A	A	N	CL
C2-05-301-10	A	A	N	CL
C2-05-337-2	A	A	N	CL
CLE234	A	A	N	CL
Cowabbie	A	A	N	CL
DVORAN	A	A	N	CL
Fleet	A	A	N	CL
Galina	A	A	N	CL
Granifen	A	A	N	CL
H92036005Z	A	A	N	CL
H96009006	A	A	N	CL
Hannan	A	A	N	CL
Hannchen	A	A	N	CL
Heart	A	A	N	CL
I91-454	A	A	N	CL
I93-608	A	A	N	CL
IGB1133	A	A	N	CL
IGV3-309	A	A	N	CL
Kustaa	A	A	N	CL
MC9939-008	A	A	N	CL
MN607	A	A	N	CL
Moondyne	A	A	N	CL
Morivian	A	A	N	CL
NRB08308	A	A	N	CL
SH99616	A	A	N	CL
SHN296	A	A	N	CL
SM060103	A	A	N	CL
SR426(M)	A	A	N	CL
SVA11	A	A	N	CL
Tremois	A	A	N	CL
WI4546	A	A	N	CL
Xanadu	A	A	N	CL

### Polymorphism of *Cly1* gene

The full length of the *Cly1* genomic DNA is 2691 bp. Several primers were used to amplify and sequence the *Cly1* gene (Table 1). Five lines with cleistogamous genotype but chasmogamous phenotype (Table 2) were investigated. By comparing the sequences of these lines, 13 SNPs were discovered in the *Cly1* gene region, of which seven were anchored to exon regions and six to intron regions (Table 3). Of the seven SNPs in exon regions, three (SNP2, SNP9, SNP12) gave rise to amino acid changes, SNP2 from Cys to Tyr, SNP9 from His to Arg, and SNP12 from Ser to Arg (Table 3). SNP10, SNP11, and SNP12 were localized in the miR172 target region reported by Nair et al. [10]. The phenotype of the accessions shown in Table 3 were chasmogamous. By comparing the genotypes and phenotypes of these lines, we can conclude that the variations in SNP1, SNP3, SNP5 –SNP10 and SNP13 are not associated with floral types.

**Table 3:** SNPs and haplotypes of the *Cly1* gene in 26 barley accessions. SNP genotypes were expressed by A, T, G or C. Empty cells indicate no polymorphism detected through next-generation sequencing or no sequence data available for the SNP.

Name	SNP 1	SNP 2	SNP 3	SNP 4	SNP 5	SNP 6	SNP 7	SNP 8	SNP 9	SNP 10	SNP 11	SNP 12	SNP 13	Haplotypes
Position (bp)	588	626	1316	1575	2071	2511	2522	2734	3044	3078	3084	3090	3188	
Intron/exon	Exon	Exon	Intron	Exon	Intron	Intron	Intron	Intron	Exon	Exon	Exon	Exon	Intron	
Amino Acid	Gly	Cys–Tyr		Asp					His–Arg	Arg	Arg	Ser–Arg		
Kinukei7	G	T	T	G	C	C	A	C	C	A	G	A	C	1
C2-05-101/263	G	T	T	G	C	C	A	C	C	A	G	A	C	1
WVB35	G	T	T	G	C	C	A	C	C	A	G	A	C	1
Langstrand0102	C	C	T	A	C	C	G	G	C	C	A	C	A	2
Prosa	C	C	T	A	C	C	G	G	C	C	A	C	A	2
Morex	C	C	C	G	G	G	G	C	T	C	A	A	A	3
Bowman	C		C		G	G	G	C	T	C	A	A	A	3
Vlamingh	C		C		G	G	G	C	T	C			A	3
W1	C		C		G	G	G	C	T	C			A	3
Haruna_Nijo	G		T		C	C	A	C	C	A			C	4
Igri	G		T		C	C	A	C	C	A	A	A	C	4
Commander	G		T		C	C	A	C	C	A	A	A	C	4
Scope	G		T		C	C	A	C	C	A	A	A	C	4
Barke	G		T		C	C	A	C	C	A	A	A	C	4
WI4304	G		T		C	C	A	C	C	A	A	A	C	4
B1k-04-12	G		T		C	C	G	C	C	A	A	A	C	4
Hindmarsh	G		T		C	C	G	C	C	A	A	A	C	4
La Trobe	G		T		C	C	G	C	C	A	A	A	C	4
Ac Metcalfe	C		T		C	C	G	C	C	A	A	A	C	4
Baudin	C		T		C	C	G	C	C	A	A	A	C	4
EC2.1	C		T		C	C	G	C	C	A	A	A	C	4
EC2.2	C		T		C	C	G	C	C	A	A	A	C	4
Fleet	C		T		C	C	G	C	C	A	A	A	C	4
EC7.1	C		T		C	C	G	G	C	C	A	A	A	4
EC7.2	C		T		C	C	G	G	C	C	A	A	A	4
X1	C		T		C	C	G	G	C	C	A	A	A	4

An additional 18 barley lines were sequenced using next-generation sequencing technology (data not shown). The sequences of Morex, Barke, and Bowman were downloaded from IPK. By aligning these lines, four haplotypes were identified in these barley accessions (Table 3).

### Correlation analysis between grain fertility percentage and temperature

**Fig 2:**
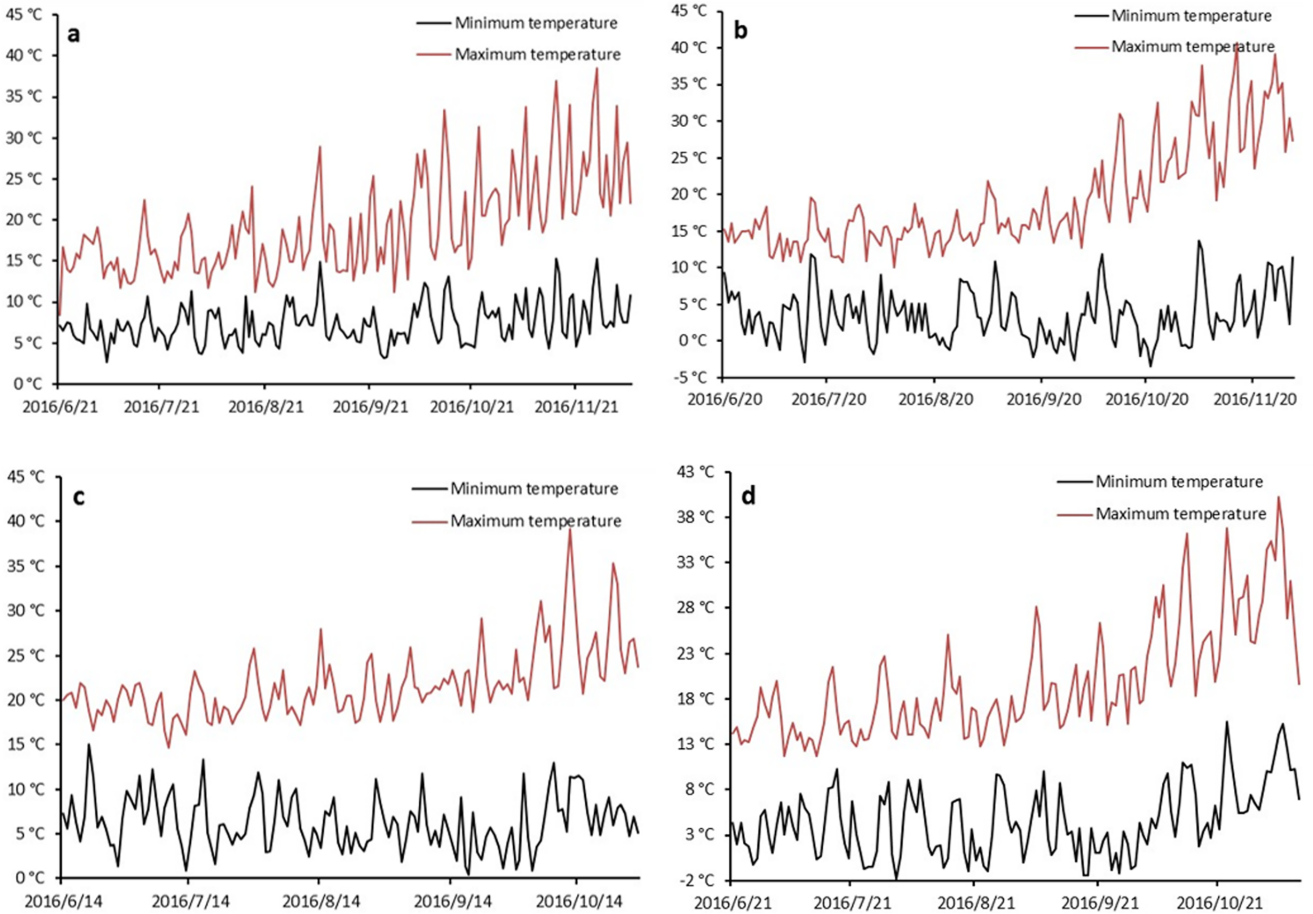
Minimum and maximum temperature curves at four locations. (a) Esperance. (b) Katanning. (c) Geraldton. (d) Merredin.

Temperature monitors were installed in four field trials to record temperature every 15 min. Temperatures were recorded for one month in Geraldton (August to September 2016) and Merredin (September to October 2016) and two months in Esperance and Katanning (September to November 2016). For the monitoring period, the respective minimum and maximum temperatures were 1.8°C and 27.9°C in Geraldton, –1.2°C and 36.2°C in Merredin, –3.4°C and 40.7°C in Katanning, and 3.2°C and 33.4°C in Esperance (Fig 2). There were nine days with the minimum temperature of below–1.0°Cin Katanning, and three days in Merredin. For heat stress, there were 11 days with the maximum temperature of above 30°C in Kattaning and nine days in Merredin. In field trials, for the temperatures, less than –1.0°Cor over 30°C would reduce fertility in barley [35,36]. We concluded that Katanning and Merredin experienced both frost and heat stress.

**Fig 3:**
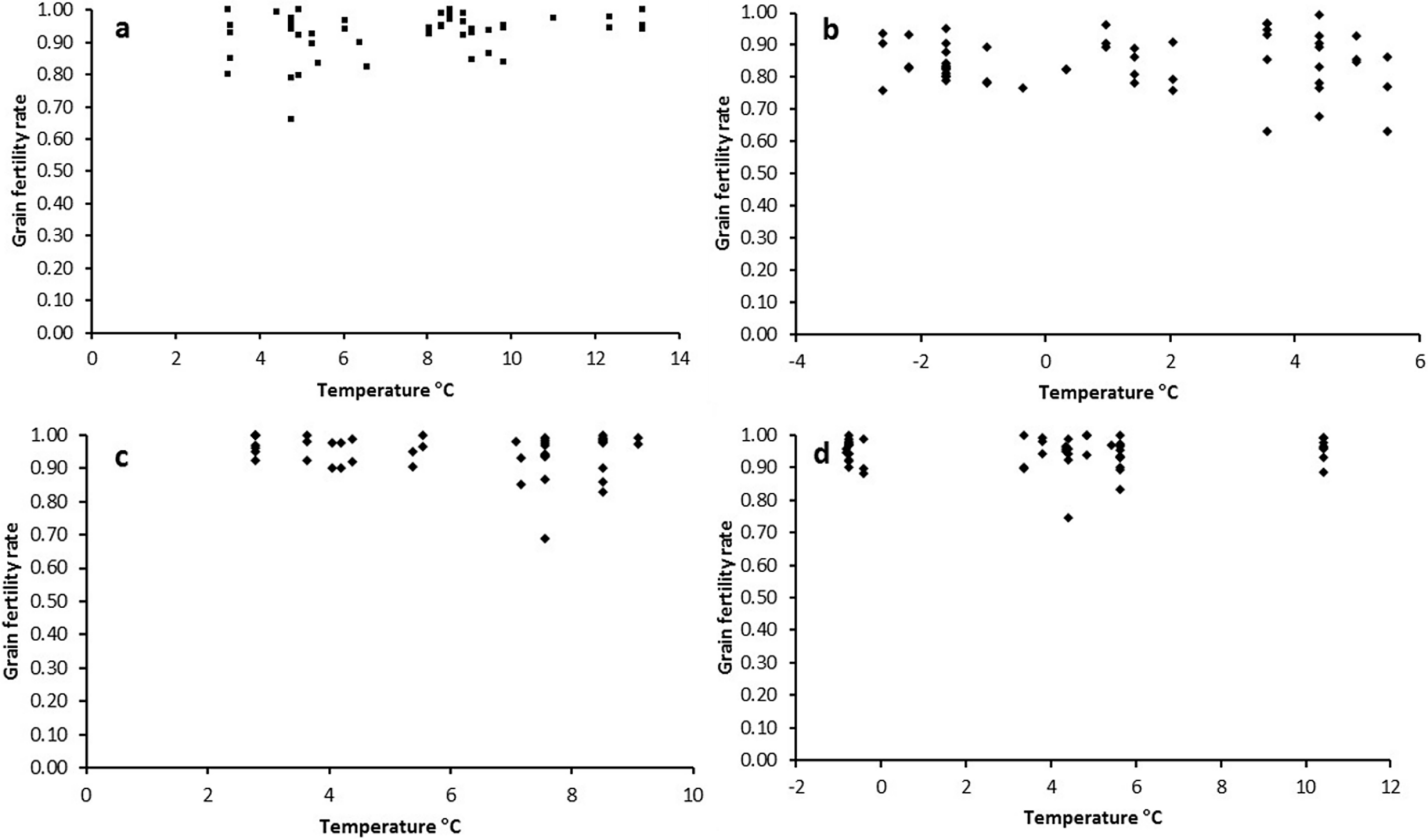
Scatter plot of grain fertility rate and intraday minimum temperature at the four locations. (a) Esperance. (b) Katanning. (c) Geraldton. (d) Merredin.

Grain fertility rates were calculated for the four trial sites (Fig 3 and Table 4). Katanning had the lowest grain fertility rate (~85%), which was significantly lower than that at Geraldton, Merredin, and Esperance (~95%). This finding is consistent with the temperature stress experienced at Katanning, being frost stress (–3.4°C) and heat stress (40.7°C).

**Table 4:** Association analysis between grain fertility rate (GFR) and floral type.

	Geraldton	Katanning	Esperance	Merredin
Cleistogamous GFR	0.940±0.066	0.850±0.076	0.921±0.061	0.943±0.048
Chasmogamous GFR	0.960±0.048	0.841±0.088	0.930±0.083	0.955±0.043
*r*-value	0.171	–0.060	0.061	0.129

Correlation coefficients were calculated for grain fertility percentage and the minimum day time temperature when the plants started to flower. The respective *r*-values for minimum and maximum temperatures were 0.293 and 0.205 in Esperance, –0.032 and –0.138 in Katanning, –0.165 and –0.208 in Geraldton, and 0.012 and 0.055 in Merredin. However,the *t*-test results indicated no clear linear relationship between grain fertility percentage and low (–3.4°C to 12°C) or high (13°C to 34°C) temperatures at flowering.

### Correlation analysis between grain fertility percentage and floral type

Barley accessions flowered at different times at each location. Flowering time ranged from 44–91 days in Geraldton, 72–131 days in Merredin, 75–131 days in Geraldton, and 72–146 days in Esperance.

Prior to the correlation analysis for floral type and grain fertility rate, chasmogamous lines with similar flowering times to cleistogamous lines were randomly selected. The *r*-values for these lines were calculated for each trial site, with Geraldton (0.171) > Merredin (0.129) > Esperance (0.061) > Katanning (–0.060) (Table 4).

### Lines with high grain fertility rate under frost stress

Three barley accessions (Wimmera, SB99252, and Torrens) had >99% grain fertility rate in Katanning where barley experienced frost and heat stress. These lines also had >95% grain fertility rate at the other three sites, except SB99252 which had 88.8% in Esperance. The barley line Torrens had a grain fertility rate of 99% in Katanning after –2.6°C frost stress during flowering, 100% in Geraldton, and 96–99% in Esperance and Merredin.

## Discussion

### SNPs in *Cly1* gene region

Nair et al. [10] analyzed the floral type and *Cly1* gene in 274 barley lines to find two SNPs correlated with cleistogamy. The SNPs were located in the miR172 target region. In situ RNA hybridization showed that both the cleistogamous and chasmogamous types presented signals. But RACE experiments indicated that miR172 guided the cleavage of *Cly1* mRNA [10]. Our study successfully developed molecular markers for these two SNPs, which were able to explain the variation in floral type in 90% of the barley accessions.

In the present study, 436 barley cultivars were characterized by *Cly1* genotype and floral type. In contrast to the study described by Nair et al. [10], we used more barley accessions. Genotyping the barley lines with molecular markers identified 391 lines with consistent genotype and phenotype and 45 lines with inconsistent genotype and phenotype. In these 45 lines, five lines showed chasmogamous type, but molecular markers indicated cleistogamous types. The other 40 lines showed the reverse genotypes and phenotypes. Environments including temperature, humid and wind may have an impact on floral type. Phenotypes will be further investigated in future trials.

There are several reasons why a chasmogamous type may convert to a cleistogamous type. In this study, only the gene region was sequenced, but the promoter region controlling the expression level may have had an impact on gene function. For example, for the barley aluminum tolerance gene *HvAACT1*, two SNPs were identified in the open reading frame region between tolerant and susceptible barley lines, but they were unrelated [37]. Gene expression results indicated that aluminum tolerance was attributed to promoter region differences. Fujii et al. [38] found a large 1-kb insertion upstream of the *HvAACT1* coding region in aluminum-tolerant accessions, which increased the gene expression level and altered the expression location to root tips.

DNA methylation in the promoter region regulates gene expression. Methylation is also found in gene bodies [39], but its role is unclear. The methylation of DNA in the *Cly1* promoter region and gene body region has not been studied but may explain the floral type of these lines. The promoter region of gene *Cly1* will be investigated in these lines with inconsistent genotype and phenotype.

Nair et al. [10] reported that two SNPs (SNP11 and SNP12 in this study) were located on the miR172 targeting site. According to Nair et al. [10], miR172 guided cleavage of the *Cly1* transcript in chasmogamous barley lines, but could not cut the *Cly1* transcript and then change floral type in cleistogamous lines. If mutations occurred in the miR172 gene region, miR172 could not interact with 21 bp of the *Cly1* gene region so the plant should exhibit a cleistogamous type. The sequence of miR172 will be investigated in future experiments.

Turuspekov et al. [21] developed ten populations from cleistogamous and chasmogamous barley parental lines. Some of the F1 crosses displayed a cleistogamous type, while others had a chasmogamous type. This indicates that cleistogamy was dominant in some lines, while chasmogamy was dominant in others. Six populations were used to map the two dominant traits. The results showed that the two dominant traits were all mapped to chromosome 2H and the same QTL region [21]. Whether the two QTLs were the same gene or different alleles is unclear.

### Grain fertility percentage and temperature

During flowering, Geraldton and Esperance had moderate temperatures, while Merredin and Katanning experienced frost stress. For example, Katanning had its minimum temperature of –3.4°C on 21 October 2016 (Fig 2), which reduced the grain fertility rate to 85% (Table 4). The other three sites had grain fertility rates from 92–96% (Table 4 and Fig 3). In Katanning, with its frost stress, Wimmera and Torrens had grain fertility rates of 99%, as well as 96–100% at the other sites; these two lines will be useful for frost stress studies in the future.

Previous studies have reported QTLs linked with frost tolerance in barley [17,40]. For example, Chen et al. [17] scored the tolerance to frost damage on reproductive tissues, and detected two QTLs for frost tolerance, one on chromosome 2H and the other on chromosome 5H [17]. A study conducted by Visioni et al. [40] identified three QTLs (2HL, 4HL, and 5HL) associated with frost tolerance using 148 barley lines. These two studies [17,40] reported a common QTL on 2HL associated with frost tolerance. SNP 11_20320 (2HL QTL) was located in a 120 cM region on chromosome 2H based on the barley POPseq [41]. The QTL for frost tolerance on chromosome 2H located in a 127 cM region was close to the cleistogamy gene *Cly1*. However, whether the cleistogamy gene *Cly1* has any impact on frost tolerance is unknown.

### Grain fertility rate and floral type

The grain fertility percentage was studied between cleistogamous and chasmogamous types at the four trial sites, with no significant differences identified between the two floral types. Furthermore, the lines with inconsistent genotype and phenotype were removed and the grain fertility percentage was calculated again (S1 Table). No significant differences in grain fertility rate were found between the two haplotypes of the cleistogamy gene *Cly1*. Genetic background of the barley accessions may have an impact on the result. Even though they were grown in the same environment and same flowering time, the grain fertility rate may differ due to genetic diversity. Therefore, the alternative way to investigate the *Cly1* gene and grain fertility rate is to use near-isogenic lines.

## Conclusions

This is the first report to investigate the relationship between grain fertility rate and floral type in barley. The trial sites at Geraldton and Esperance hadmoderate temperatures during flowering, while those at Merredin and Katanning experienced frost and heat stress. Frost stress reduced the grain fertility rate. Two barley lines, Wimmera and Torrens, with high grain fertility rates under frost stress, would be useful for frost stress studies in barley. Thirteen SNPs were detected in the *Cly1* gene region across 436 barley accessions (Table 3), and four haplotypes were identified.

Association analysis between grain fertility rate and cleistogamous and chasmogamous barley lines was inconclusive. Both the environment and genetic background influence grain fertility. Near-isogenic lines will be used to evaluate their relationship between thermal tolerance and floral type further.

## Supporting information

S1 Fig Four trial locations were shown in the geographic map. (TIF)

S2 Fig The specific primers used in this study were shown in *Cly1* sequence. (TIF)

S3 Fig The representative photo of floral type in barley. Left: cleistogamy. Right: chasmogamy.
(TIF)

S1 Table Association analysis between grain fertility rate (GFR) and genotypes of *Cly1*. (DOCX)

## References

[1] Karsai I, Igartua E, Casas AM, Kiss T, Soos V, Balla K, et al (2013) Developmental patterns of a large set of barley (*Hordeum vulgare*) cultivars in response to ambient temperature. Annals of Applied Biology 162: 309–323.

[2] Lobell DB, Field CB (2007) Global scale climate crop yield relationships and the impacts of recent warming. Environmental Research Letters 2.

[3] Calderini DF, Savin R, Abeledo LG, Reynolds MP, Slafer GA (2001) The importance of the period immediately preceding anthesis for grain weight determination in wheat. Euphytica 119: 199–204.

[4] Matsui T, Kagata H (2003) Characteristics of floral organs related to reliable self-pollination in rice (*Oryza sativa L*.). Ann Bot 91: 473–477. doi: 10.1093/aob/mcg045 12588727 10.1093/aob/mcg045 PMC4241067

[5] Koike S, Yamaguchi T, Ohmori S, Hayashi T, Yatou O, Yoshida H (2015) Cleistogamy decreases the effect of high temperature stress at flowering in rice. Plant Production Science 18: 111–117.

[6] Culley TM, Klooster MR (2007) The cleistogamous breeding system: a review of its frequency, evolution, and ecology in angiosperms. Botanical Review 73: 1–30.

[7] Cherplick GP (2007) Plasticity of chasmogamous and cleistogamous reproductive allocation in grasses. Aliso: A Journal of Systematic and Evolutionary Botany 23: 286–294.

[8] Peter CI, Johnson SD (2006) Anther cap retention prevents self-pollination by elaterid beetles in the South African orchid *Eulophia foliosa*. Annals of Botany 97: 345–355. doi: 10.1093/aob/mcj041 16373371 10.1093/aob/mcj041 PMC2803648

[9] Culley TM (2002) Reproductive biology and delayed selfing in *Viola pubescens* (Violaceae), an understory herb with chasmogamous and cleistogamous flowers. International Journal of Plant Sciences 163: 113–122.

[10] Nair SK, Wang N, Turuspekov Y, Pourkheirandish M, Sinsuwongwat S, Chen G, et al (2010) Cleistogamous flowering in barley arises from the suppression of microRNA-guided HvAP2 mRNA cleavage. Proceedings of the National Academy of Sciences of the United States of America 107: 490–495. doi: 10.1073/pnas.0909097107 20018663 10.1073/pnas.0909097107 PMC2806734

[11] Abdel-Ghani AH, Parzies HK, Omary A, Geiger HH (2004) Estimating the outcrossing rate of barley landraces and wild barley populations collected from ecologically different regions of Jordan. Theoretical and Applied Genetics 109: 588–595. doi: 10.1007/s00122-004-1657-1 15083273 10.1007/s00122-004-1657-1

[12] Dahleen LS, Morgan W, Mittal S, Bregitzer P, Brown RH, Hill NS (2012) Quantitative trait loci (QTL) for *Fusarium* ELISA compared to QTL for Fusarium head blight resistance and deoxynivalenol content in barley. Plant Breeding 131: 237–243.

[13] Daniell H (2002) Molecular strategies for gene containment in transgenic crops. Nature Biotechnology 20: 843–843. 10.1038/nbt0602-581 PMC3471138 12042861

[14] Hori K, Sato K, Kobayashi T, Takeda K (2006) QTL analysis of fusarium head blight severity in recombinant inbred population derived from a cross between two-rowed barley varieties. Breeding Science 56: 25–30.

[15] Ma SM, Wang YF (2004) Molecular strategies for decreasing the gene flow of transgenic plants. Yi Chuan 26: 556–559. 15640061

[16] Wang N, Ning SZ, Pourkheirandish M, Honda I, Komatsuda T (2013) An alternative mechanism for cleistogamy in barley. Theoretical and Applied Genetics 126: 2753–2762. doi: 10.1007/s00122-013-2169-7 23925483 10.1007/s00122-013-2169-7

[17] Chen A, Reinheimer J, Brule-Babel A, Baumann U, Pallotta M, Fincher G, et al (2009) Genes and traits associated with chromosome 2H and 5H regions controlling sensitivity of reproductive tissues to frost in barley. Theoretical and Applied Genetics 118: 1465–1476. doi: 10.1007/s00122-009-0995-4 19277599 10.1007/s00122-009-0995-4

[18] Honda I, Turuspekov Y, Komatsuda T, Watanabe Y (2005) Morphological and physiological analysis of cleistogamy in barley (*Hordeum vulgare*). Physiologia Plantarum 124: 524–531.

[19] Johansen B, Vonbothmer R (1994) Pollen size in *Hordeum* L- correlation between size, ploidy level, and breeding system. Sexual Plant Reproduction 7: 259–263.

[20] Morinaga S, Nagano AJ, Miyazaki S, Kubo M, Demura T, Fukuda TDH, et al (2008) Ecogenomics of cleistogamous and chasmogamous flowering: genome wide gene expression patterns from cross-species microarray analysis in *Cardamine kokaiensis* (Brassicaceae). Journal of Ecology 96: 1086–1097.

[21] Turuspekov Y, Mano Y, Honda I, Kawada N, Watanabe Y, Komatsuda T (2004) Identification and mapping of cleistogamy genes in barley. Theoretical and Applied Genetics 109: 480–487. doi: 10.1007/s00122-004-1673-1 15138690 10.1007/s00122-004-1673-1

[22] Chhabra AK, Sethi SK (1991) Inheritance of cleistogamic flowering in durum wheat (*Triticum Durum*). Euphytica 55: 147–150.

[23] Merwine NC, Gourley LM, Blackwell KH (1981) Inheritance of papery glume and cleistogamy in sorghum. Crop Science 21: 953–956.

[24] Takahashi R, Kurosaki H, Yumoto S, Han OK, Abe J (2001) Genetic and linkage analysis of cleistogamy in soybean. Journal of Heredity 92: 89–92. 11336238 10.1093/jhered/92.1.89

[25] Yoshida H, Itoh JI, Ohmori S, Miyoshi K, Horigome A, Uchida E, et al (2007) *Superwoman1-cleistogamy*, a hopeful allele for gene containment in GM rice. Plant Biotechnology Journal 5: 835–846. doi: 10.1111/j.1467-7652.2007.00291.x 17764519 10.1111/j.1467-7652.2007.00291.x

[26] Wang N, Ning SZ, Wu JZ, Tagiri A, Komatsuda T (2015) An epiallele at *cly1* affects the expression of floret closing (cleistogamy) in barley. Genetics 199: 95–104. doi: 10.1534/genetics.114.171652 25398791 10.1534/genetics.114.171652 PMC4286696

[27] Liu XQ, Rong JY, Liu XY (2008) Best linear unbiased prediction for linear combinations in general mixed linear models. Journal of Multivariate Analysis 99: 1503–1517.

[28] Billadeau D, Blackstadt M, Greipp P, Kyle RA, Oken MM, Kay N, et al (1991) Analysis of B-lymphoid malignancies using allele specific polymerase chain reaction—a technique for sequential quantitation of residual disease. Blood 78: 3021–3029. 1954387

[29] Howard PL, Collins CC, Heintz NH (1991) Polymerase chain reaction and allele specific oligonucleotides in paternity testing of the deceased. Transfusion 31: 441–442. 1675501 10.1046/j.1537-2995.1991.31591263200.x

[30] Lo ESF, Lo YMD, Tse CH, Fleming KA (1991) An allele specific polymerase chain reaction assay to detect hepatitis B precore mutants in fulminant hepatitis-B. Hepatology 14: 66.

[31] Pinder SJ, Perry BN, Savva D, Skidmore CJ (1990) The polymerase chain reaction applied to identification of specific alleles of the bovine milk protein genes. Biochemical Society Transactions 18: 675–676. 1980469 10.1042/bst0180675

[32] Sarkar G, Cassady J, Bottema CDK, Sommer SS (1990) Characterization of polymerase chain reaction amplification of specific alleles. Anal Biochem 186: 64–68. 2192583 10.1016/0003-2697(90)90573-r

[33] Silverman LM, Hinshaw MT, Friedman KJ, Highsmith WE (1991) An optimized protocol for the detection of delta F508 by allele specific polymerase chain reaction. American Journal of Human Genetics 49: 204–204.

[34] Suzuki Y, Sekiya T, Hayashi K (1991) Allele specific polymerase chain reaction—a method for amplification and sequence determination of a single component among a mixture of sequence variants. Anal Biochem 192: 82–84. 2048738 10.1016/0003-2697(91)90188-y

[35] Reinheimer JL, Barr AR, Eglinton JK (2004) QTL mapping of chromosomal regions conferring reproductive frost tolerance in barley (*Hordeum vulgare* L.). Theoretical and Applied Genetics 109: 1267–1274. doi: 10.1007/s00122-004-1736-3 15365623 10.1007/s00122-004-1736-3

[36] Abiko M, Akibayashi K, Sakata T, Kimura M, Kihara M, Itoh K, et al (2005) High-temperature induction of male sterility during barley (*Hordeum vulgare* L.) anther development is mediated by transcriptional inhibition. Sexual Plant Reproduction 18: 91–100.

[37] Furukawa J, Yamaji N, Wang H, Mitani N, Murata Y, Sato K, et al (2007) An aluminum-activated citrate transporter in barley. Plant and Cell Physiology 48: 1081–1091. doi: 10.1093/pcp/pcm091 17634181 10.1093/pcp/pcm091

[38] Fujii M, Yokosho K, Yamaji N, Saisho D, Yamane M, Takahshi H, et al (2012) Acquisition of aluminium tolerance by modification of a single gene in barley. Nature Communications 3. 10.1038/ncomms1726 PMC3316887 22395604

[39] Ball MP, Li JB, Gao Y, Lee JH, LeProust EM, Park IH, et al (2009) Targeted and genome-scale strategies reveal gene-body methylation signatures in human cells Nature Biotechnology 27: 485–485. 10.1038/nbt.1533 PMC3566772 19329998

[40] Visioni A, Tondelli A, Francia E, Pswarayi A, Malosetti M, Russell J, et al (2013) Genome-wide association mapping of frost tolerance in barley (*Hordeum vulgare* L.). BMC Genomics 14. 23802597 10.1186/1471-2164-14-424 PMC3701572

[41] Mascher M, Muehlbauer GJ, Rokhsar DS, Chapman J, Schmutz J, Barry K, et al (2013) Anchoring and ordering NGS contig assemblies by population sequencing (POPSEQ). Plant Journal 76: 718–727. doi: 10.1111/tpj.12319 23998490 10.1111/tpj.12319 PMC4298792

