# Supplementary material for: Polymorphism of floral type gene Cly1 and its association with thermal stress in barley
Source: PLoS One. 2018 Mar 1;13(3):e0193390. doi: 10.1371/journal.pone.0193390 (PMC5832248; doi:10.1371/journal.pone.0193390)
Supplement: S1 Table — (DOCX) [file pone.0193390.s004.docx]

S1 Table. Association analysis between grain fertility rate (GFR) and genotypes of *Cly1*

|  | Geraldton | Katanning | Esperance | Merredin |
| --- | --- | --- | --- | --- |
| Cleistogamous | 0.960±0.051 | 0.837±0.092 | 0.926±0.086 | 0.957±0.044 |
| Chasmogamous | 0.934±0.075 | 0.849±0.076 | 0.924±0.061 | 0.939±0.049 |
| *r*-value | 0.197 | 0.073 | 0.009 | 0.185 |
